# Meta-analysis of 16S rRNA microbial data identified alterations of the gut microbiota in COVID-19 patients during the acute and recovery phases

**DOI:** 10.1186/s12866-022-02686-9

**Published:** 2022-11-14

**Authors:** Xiaomin Cheng, Yali Zhang, Yifan Li, Qin Wu, Jiani Wu, Soo-Kyung Park, Cheng Guo, Jiahai Lu

**Affiliations:** 1 School of Public Health, Sun Yat-sen University, Guangzhou, 510080 Guangdong Province China; 2 Guangzhou Nansha District Center for Disease Control and Prevention, Guangzhou, China; 3 Division of Gastroenterology, Department of Internal Medicine and Inflammatory Bowel Disease Center, Kangbuk Samsung Hospital, Sungkyunkwan University School of Medicine, Seoul, Korea; 4 Center for Infection and Immunity, Mailman School of Public Health, Columbia University, New York, NY 10032 USA; 5 One Health Center of Excellence for Research and Training, School of Public Health, Sun Yat-sen University, Guangzhou, China; 6 NMPA Key Laboratory for Quality Monitoring and Evaluation of Vaccines and Biological Products, Guangzhou, China; 7 Key Laboratory for Tropical Disease Control, Ministry of Education, Guangzhou, China; 8 Research Institute of Sun Yat-Sen University in Shenzhen, Shenzhen, China; 9 One Health Research Center, Hainan Medical University, Haikou, 571199 China

**Keywords:** COVID-19, Gut microbiota, Acute phase, Recovery phase, 16S rRNA

## Abstract

**Background:**

Dozens of studies have demonstrated gut dysbiosis in COVID-19 patients during the acute and recovery phases. However, a consensus on the specific COVID-19 associated bacteria is missing. In this study, we performed a meta-analysis to explore whether robust and reproducible alterations in the gut microbiota of COVID-19 patients exist across different populations.

**Methods:**

A systematic review was conducted for studies published prior to May 2022 in electronic databases. After review, we included 16 studies that comparing the gut microbiota in COVID-19 patients to those of controls. The 16S rRNA sequence data of these studies were then re-analyzed using a standardized workflow and synthesized by meta-analysis.

**Results:**

We found that gut bacterial diversity of COVID-19 patients in both the acute and recovery phases was consistently lower than non-COVID-19 individuals. Microbial differential abundance analysis showed depletion of anti-inflammatory butyrate-producing bacteria and enrichment of taxa with pro-inflammatory properties in COVID-19 patients during the acute phase compared to non-COVID-19 individuals. Analysis of microbial communities showed that the gut microbiota of COVID-19 recovered patients were still in unhealthy ecostates.

**Conclusions:**

Our results provided a comprehensive synthesis to better understand gut microbial perturbations associated with COVID-19 and identified underlying biomarkers for microbiome-based diagnostics and therapeutics.

**Supplementary Information:**

The online version contains supplementary material available at 10.1186/s12866-022-02686-9.

## Introduction

The pandemic of coronavirus disease 2019 (COVID-19) is a global health issue caused by infection from the severe acute respiratory syndrome coronavirus 2 (SARS-CoV-2), which has devastated economies and overwhelmed healthcare systems all over the world [1]. Moreover, the emergence and rapid spread of various SARS-CoV-2 variants of concern (VOCs) and variants of interest (VOIs) that is more contagious and potential to evade immunity has posed challenges to the control of the COVID-19 pandemic [2, 3]. It is essential to explore disease pathogenesis and develop new therapeutic strategies for COVID-19. Evidence accumulating in humans and animals implicated that gut dysbiosis is associated with infectious diseases caused by viral infections, such as influenza, HIV infection, and Chikungunya virus (CHIKV) infection [4–6]. Association between gut dysbiosis and SARS-CoV-2 infection has been receiving increasing attention [7]. Most COVID-19 patients exhibit gastrointestinal symptoms and inflammation [8, 9]. The possibility that gut dysbiosis contributes to SARS-CoV-2 infection was also raised by animal study, showing that the microbiota can affect angiotensin-converting enzyme 2 (ACE2) [10]. It has been extensively known that ACE2 is regarded as the crucial receptor for cell entry of SARS-CoV-2, which is widely distributed in alveolar tissue and gut [11–14]. Further, gut microbial alteration was reported to be strongly associated with persistent symptoms in COVID-19 [15]. It has been reported that known probiotic supplementation can promote symptom resolution and viral clearance in COVID-19 patients [16, 17]. These suggest pivotal roles of gut microbiota in developing diagnostic biomarkers and therapeutic strategies for COVID-19.

Human studies from different populations have investigated perturbations in gut microbiota in COVID-19 patients during the acute and recovery phases through 16S rRNA gene amplified sequencing [17–39]. The common conclusion across these studies is that the gut microbiota of COVID-19 patients differs from that of healthy subjects. However, most of these studies are limited by small sample sizes, and, particularly, have yielded plentiful inconsistent results on the bacterial taxa associated with the disease. The wide variation of the gut microbiota across different populations, lifestyles, and diets together with heterogeneity in study designs, data processing and statistical methods make these reported results difficult to interpret [40]. Meta-analyses have been proposed to solve these problem for providing an effective approach to screen for consistent disease-associated microbial signatures, especially specific bacterial taxa, which may be promising biomarkers enhancing diagnostic accuracy, guiding treatment, promoting the monitoring of prognosis and improving the efficacy of vaccinations [41–43]. Here, we conducted a systematic review and meta-analysis of published studies that reported 16S rRNA gene sequences of fecal samples or rectal swabs from adult patients with COVID-19. We reanalyzed the 16S rRNA sequence data through a uniform analysis pipeline to identify the robust and reproducible perturbations in gut microbiota and elucidate potential microbial biomarkers in COVID-19.

## Results

### Study selection

The details of the searching and screening process were summarized in a flowchart (Fig. S1). After review, we identified 23 relevant articles, of which, 16 eligible articles were re-analyzed in our study (Table 1 and Table S2 showed information of studies included and excluded, separately). Ten studies were from Asia, others from America and Europe. The Illumina MiSeq was the most widely used sequencing platform (*n* = 11). The analysed datasets were generated mostly based on the V3–V4 regions of the 16S rRNA gene (*n* = 14). Twelve studies made a comparison of gut microbiota between COVID-19 patients (COV) and non-COVID-19 individuals (non-COV); six made a comparison between COVID-19 recovered patients (RP/post-RP) and non-COV; four made a comparison between COV and RP/post-RP; and four studies involved gut microbiota of COVID-19 patients with different severity levels, including 161 non-severe COVID-19 patients and 108 severe COVID-19 patients. A total of 1385 individual samples were available, including 697 COV, 557 non-COV, 79 RP, and 52 post-RP. In most studies (*n* = 9), age and gender were not significantly different among the groups. Fourteen studies assessed the differential abundance of gut bacterial taxa or pathways between groups, of which, 11 studies were conducted without adjustment for relevant covariates, such as age, gender, antibiotics treatment, and so on.

**Table 1:** Characteristics of Studies Included

Author, Year [Ref]	Country	Design	Study population ^***a***^	Sample type	16S region	Sequencing platform	Differential taxa analysis
Al 2021 [18]	United Arab Emirates	Case-control	78 COV, 50 non-COV	Fecal samples	COV: V3-V4; non-COV: V4	MiSeq	DESeq2
Cervino 2022 [19]	France	Case-control	55 COV, 76 non-COV	Fecal samples	V3-V4	MiSeq	Wilcoxon test
Chen 2022 [26]	China	Longitudinal	26 COV, 20 RP, 30 post-RP, 30 non-COV	Fecal samples	V3-V4	MiSeq	NM
Gaibani 2021 [27]	Italy	Case-control	69 COV, 69 non-COV	Fecal samples	COV: V3-V4; non-COV: V3-V4, V1-V3	MiSeq, 454 GS Junior	LEfSe
Gu 2020 [28]	China	Case-control	30 COV, 30 non-COV	Fecal samples	V3-V4	MiSeq	LEfSe
Khan 2021 [29]	India	Case-control	30 COV (10 asymptomatic, 10 non-severe, 10 severe), 10 non-COV	Fecal samples	V3-V4	HiSeq 2000	LEfSe
Kim 2021 [30]	Korea	Longitudinal	12 COV, 12 RP, 36 non-COV ^*b*^	Fecal samples	V3–V4	MiSeq	MaAsLin2
Mazzarelli 2021 [31]	Italy	Case-control	15 COV, 8 non-COV	Rectal swabs	V2, V4, V8, V3-6, 7-9	Ion Torrent S5	DESeq2
Moreira-Rosario 2021 [32]	Portugal	Cross-sectional	111 COV (54 non-severe, 57 severe)	Fecal samples	V3-V4	Ion Torrent S5	NM
Newsome 2021 [33]	USA	Case-control	50 COV, 9 RP, 34 non-COV	Fecal samples	V1-V3	MiSeq	edgeR
Rafiqul 2022 [20]	Bangladesh	Case-control	44 COV, 30 non-COV	Fecal samples	V3-V4	MiSeq	Kruskal-Wallis
Reinold 2021 [21]	Germany	Case-control	117 COV (79 non-severe, 38 severe), 95 non-COV	Rectal swabs	V3-V4	NovaSeq 6000	LEfSe
Ren 2021 [22]	China	Case-control	36 COV, 18 RP, 72 non-COV	Fecal samples	V3-V4	MiSeq	LEfSe
Tian 2021 [23]	China	Case-control	7 post-RP, 7 non-COV	Fecal samples	V3-V4	MiSeq	LEfSe
Wu 2021 [24]	China	Case-control	24 COV (21 non-severe, 3 severe; 50 samples), 20 RP (31 samples), 32 non-COV (32 samples)	Fecal samples	V3-V4	NovaSeq 6000	LEfSe/MaAslin2
Zhou 2021 [25]	China	Case-control	15 post-RP, 14 non-COV	Fecal samples	V3-V4	MiSeq	Wilcoxon test

*NM* Not mentioned. ^a^ We focused on only study subjects that have available stool samples or rectal swabs. ^b^ Only the data of the COV group and the RP group was obtained from the corresponding author, the data of the non-COV group belong to other authors

### Microbial diversity was decreased in COV

**Fig. 1:**
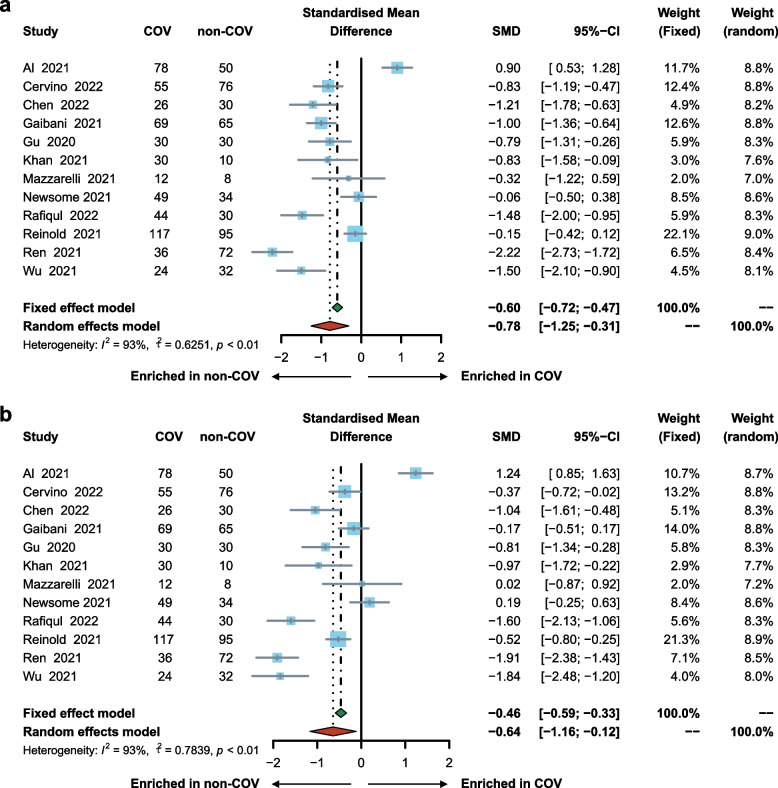
Forest plot showing differences in alpha diversity between COV and non-COV by (**a**) Shannon’s diversity index, and (**b**) observed species. COV, samples derived from COVID-19 patients; non-COV, samples derived from non-COVID-19 individuals

The pooled estimate by random effects model showed a significant decrease in COV compared to non-COV in the measures of alpha diversity, including Shannon’s diversity index (SMD = − 0.78; 95% CI, − 1.25 to − 0.31; Fig. 1a), observed species (SMD = − 0.64; 95% CI, − 1.16 to − 0.12; Fig. 1b), Pielou’s evenness (SMD = − 0.72; 95% CI, − 1.10 to − 0.34), and Faith’s phylogenetic diversity (SMD = − 0.49; 95% CI, − 0.88 to − 0.10; Fig. S2). Sensitivity analysis showed that the results were consistent after the removal of either of the included studies (Fig. S3). The funnel plots symmetry in consonance with Begg’s and Egger’s test indicated no publication bias (Fig. S4). In the subset of four studies that involved COVID-19 patients with different severity levels (one study removed due to small sample size of severe COVID-19 patients), the pooled estimate of alpha diversity by fixed effects model revealed a non-significant decrease in severe COVID-19 patients compared with non-severe COVID-19 patients as measured by Shannon’s diversity index (SMD = − 0.24; 95% CI, − 0.49 to 0.02), observed species (SMD = − 0.21; 95% CI, − 0.47 to 0.05), Pielou’s evenness (SMD = − 0.21; 95% CI, − 0.47 to 0.04), and Faith’s phylogenetic diversity (SMD = − 0.16; 95% CI, − 0.42 to 0.10; Fig. S5).

### Microbial diversity was decreased in RP/post-RP vs. non-COV

**Fig. 2:**
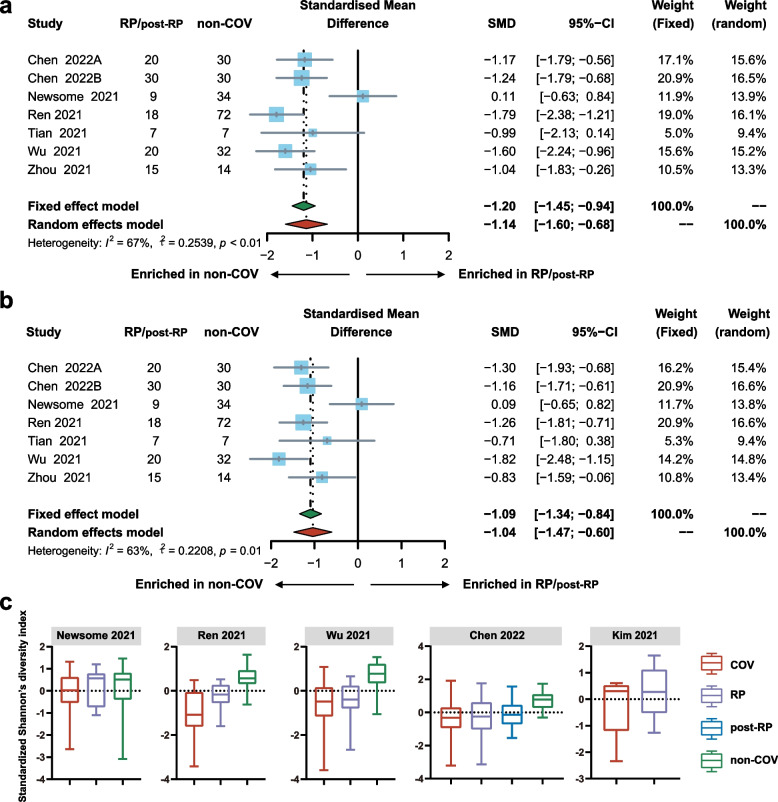
Differences in alpha diversity between RP/post-RP and non-COV. (**a**) Forest plot of the differences in Shannon’s diversity index between RP/post-RP and non-COV. (**b**) Forest plot of the differences in observed species between RP/post-RP and non-COV. (**c**) Boxplots showing standardized Shannon’s diversity index by SARS-CoV-2 infection status (red = COV, purple = RP, blue = post-RP, and green = non-COV). COV, samples derived from COVID-19 patients; RP, samples derived from COVID-19 recovered patients who with clearance of SARS-CoV-2 RNA (negative conversion of viral RNA) within one month; those with negative conversion of viral RNA more than three months were especially grouped as post-RP; non-COV, samples derived from non-COVID-19 individuals

A significant decrease in RP/post-RP compared with non-COV was demonstrated by the pooled estimate of alpha diversity using random effects model, including Shannon’s diversity index (SMD = − 1.14; 95% CI, − 1.60 to − 0.68; Fig. 2a), observed species (SMD = − 1.04; 95% CI, − 1.47 to − 0.60; Fig. 2b), Pielou’s evenness (SMD = − 0.94; 95% CI, − 1.37 to − 0.52), and Faith’s phylogenetic diversity (SMD = − 0.80; 95% CI, − 1.21 to − 0.39; Fig. S6). Sensitivity analysis showed that the results remained unchanged after the removal of one study at a time (Fig. S7). There was no publication bias identified by the funnel plots symmetry (Fig. S8). Test for subgroup differences demonstrated no significance when studies stratified by RP and post-RP (Fig. S9). Boxplots of standardized Shannon’s diversity index revealed an increased trend in RP/post-RP compared with COV (Fig. 2c). Boxplots of standardized observed species, standardized Pielou’s evenness and standardized Faith’s phylogenetic diversity demonstrated the same trend within most, though not all individual studies (Fig. S10).

### Microbial composition was altered in COV

**Fig. 3:**
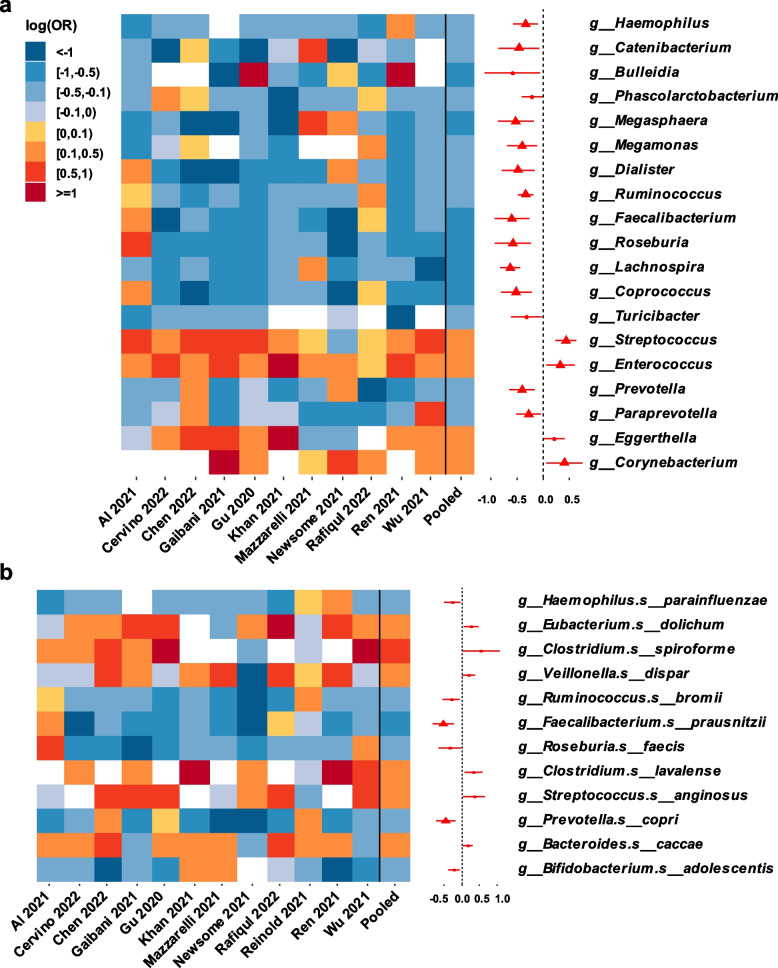
Differential gut microbial composition between COV and non-COV (**a**) at genus level, and (**b**) at species level. Heatmap showed log (OR) of relative abundances of bacterial taxa between COV and non-COV across each study. The bacterial taxa unavailable in a particular study were in white in heatmap. Forest plot indicated pooled log (OR) estimate and 95% CI of relative abundances of bacterial taxa between COV and non-COV across all studies included. Log (OR) estimates were from GAMLSS-BEZI and Random Effects Meta-analysis. Only bacterial taxa with pooled *P* of pooled log (OR) estimates below 0.05 were displayed. Pooled log (OR) estimates with FDR-adjusted pooled *P* < 0.1 were showed as red triangles. Log (OR) > 0 denoted an increase and log (OR) < 0 denoted a decrease of taxa relative abundance in COV compared with non-COV. COV, samples derived from COVID-19 patients; non-COV, samples derived from non-COVID-19 individuals

In a subset of twelve studies reporting the comparison of gut microbiota between COV and non-COV (one study removed as the fit did not converge), at the genus level, COV had significantly lower relative abundances of *Haemophilus*, *Catenibacterium*, *Megasphaera*, *Megamonas*, *Dialister*, *Ruminococcus*, *Faecalibacterium*, *Roseburia*, *Lachnospira*, *Coprococcus*, *Prevotella* and *Paraprevotella*, as well as significantly higher relative abundances of *Streptococcus*, *Enterococcus*, *Corynebacterium* as compared to non-COV in the pooled log (OR) estimate from a random-effects meta-analysis (all FDR-adjusted *P* < 0.1; Fig. 3a, Table S3). In a subset of twelve studies, at the species level, COV had significantly lower relative abundances of *Faecalibacterium prausnitzii* and *Prevotella copri* as compared to non-COV in the pooled log (OR) estimate (all FDR-adjusted *P* < 0.1; Fig. 3b, Table S3). The results remained similar in sensitivity analyses when excluding either of the included studies (data not shown). To examine whether these differences were driven mostly by studies from China, we also removed the four Chinese studies in the sensitivity analysis, though a few aforementioned genera were not significant after adjustment for multiple comparisons, the results without adjustment for multiple comparisons did not change substantially (Fig. S11 and Table S4). This suggested that our results were robust to a certain extent, as a strict adjustment for multiple comparisons is not required at all times [44]. In a subset of three studies with available data on COVID-19 patients with different severity levels, at the genus level, the relative abundances of *Roseburia* (log (OR) = − 0.31; *P* = 0.018; FDR-adjusted *P* = 0.962) and *Faecalibacterium* (log (OR) = − 0.49; *P* = 0.046; FDR-adjusted *P* = 0.962) showed a non-significant decrease in severe COVID-19 patients compared with non-severe COVID-19 patients after adjusting for multiple testing (Table S5).

### Microbial composition was altered in RP/post-RP

**Fig. 4:**
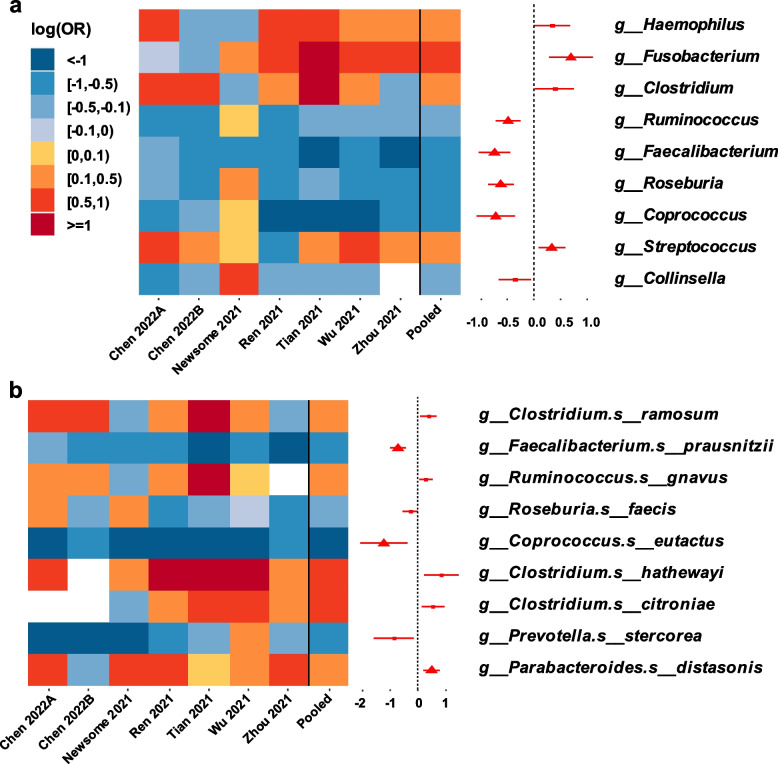
Differential gut microbial composition between RP/post-RP and non-COV (**a**) at genus level, and (**b**) at species level. Heatmap showed log (OR) of relative abundances of bacterial taxa between RP/post-RP and non-COV across each study. The bacterial taxa unavailable in a particular study were in white in heatmap. Forest plot indicated pooled log (OR) estimate and 95% CI of relative abundances of bacterial taxa between RP/post-RP and non-COV across all studies included. Log (OR) estimates were from GAMLSS-BEZI and Random Effects Meta-analysis. Only bacterial taxa with pooled *P* of pooled log (OR) estimates below 0.05 were displayed. Pooled log (OR) estimates with FDR-adjusted pooled *P* < 0.1 were showed as red triangles. Log (OR) > 0 denoted an increase and log (OR) < 0 denoted a decrease of taxa relative abundance in RP/post-RP compared with non-COV. RP, samples derived from COVID-19 recovered patients who with clearance of SARS-CoV-2 RNA (negative conversion of viral RNA) within one month; those with negative conversion of viral RNA more than three months were especially grouped as post-RP; non-COV, samples derived from non-COVID-19 individuals

In a subset of six studies (Chen 2022 study [26] involved both RP dataset and post-RP dataset), at the genus level, the relative abundances of *Ruminococcus*, *Faecalibacterium*, *Roseburia* and *Coprococcus* were found to be significantly decreased, together with the relative abundances of *Fusobacterium* and *Streptococcus* were found to be significantly increased in RP/post-RP compared with non-COV in the pooled log (OR) estimate from a random-effects meta-analysis (all FDR-adjusted *P* < 0.1; Fig. 4a, Table S6). At the species level, the relative abundances of *Faecalibacterium prausnitzii* and *Coprococcus eutactus* were significantly decreased, while the relative abundance of *Parabacteroides distasonis* was significantly increased in RP/post-RP compared with non-COV in the pooled log (OR) estimate (all FDR-adjusted *P* < 0.1; Fig. 4b). Results did not change substantially in sensitivity analyses when excluding either of the included studies (data not shown). Fewer alterations were observed in the gut microbiota between COV and RP/post-RP at the genus level. At the species level, *Clostridium clostridioforme* was detected to be significantly decreased and *Bifidobacterium breve* was detected to be significantly increased in COV as compared to RP/post-RP (Table S7).

## Discussion

Our meta-analysis revealed consistent alterations in gut microbial diversity and microbial composition in COVID-19 patients during the acute and recovery phases. Owing to the larger datasets composed of populations from different geographic regions and the consistent method workflow for analyses, our meta-analysis provides more precise estimates of the potential effects and more robust results than a single study.

Our results showed substantial gut microbiota shifts in COVID-19 patients during the acute phase. Most consistently, a significant decrease in the gut microbiota of COVID-19 patients was observed as measured by four commonly applied alpha diversity indices. Decreased diversity in gut microbiota has frequently been considered as a hallmark of diseases, such as HIV infection, recurrent *Clostridioides difficile* infection, inflammatory bowel disease [45–47]. In addition, decreased diversity can predict mortality in hematopoietic stem cell recipients [48]. High heterogeneity was found in alpha diversity analyses of COV vs. non-COV. However, sensitivity analyses showed similar findings, and our meta-analysis results in alpha diversity were roughly concordant with the original researches, implying that our results are robust. We further demonstrated alterations in gut microbial composition of COVID-19 patients. The genera *Megasphaera*, *Dialister*, *Ruminococcus*, *Faecalibacterium*, *Roseburia*, *Lachnospira*, and *Prevotella* were decreased in COVID-19 patients. As these genera are the butyrate-producing genera [19, 49, 50], the associations between gut dysbiosis and SARS-CoV-2 infection may be partly mediated by short-chain fatty acids (SCFAs), mainly acetate, propionate and butyrate, which play vital roles in maintaining mucosal integrity and exerting anti-inflammatory effects via macrophage function and down-regulation of pro-inflammatory cytokines [49, 51, 52]. Likewise, lower abundances of *Faecalibacterium*, *Dialister*, and *Lachnospira* were also observed in COVID-19 patients in a metagenomic study [53]. A previous systematic review reported that a reduction in abundances of *Faecalibacterium*, *Ruminococcus*, and *Prevotella* was associated with higher systemic inflammation characterized by increased high sensitivity C-reactive protein (hsCRP) and IL-6 [54]. At the species level, significant decreases of *F. prausnitzii* and *P. copri* were observed in COVID-19 patients in our meta-analysis, which is consistent with several metagenomic studies [53, 55, 56]. *F. prausnitzii*, known for its anti-inflammatory properties, can down-regulate the expression of pro-inflammatory cytokines like IL-6, TNF-α, and TNF-β through multiple pathways [57–59]. One metagenomic study reported that the association bewteen higher abundance of *P. copri* and less vaccine adverse events is likely mediated via their anti-inflammatory properties, suggesting a beneficial role of *P. copri* in host immune homeostasis [60]. In addition, our meta-analysis demonstrated that the opportunistic pathogens *Streptococcus*, *Enterococcus*, and *Corynebacterium* were enriched in COVID-19 patients as compared to non-COVID-19 individuals. *Streptococcus* was associated with increased expression of pro-inflammatory cytokines such as IL-18, TNF-α, and IFN-γ [61]. Pro-inflammatory cytokines may in turn predispose host to gut dysbiosis and consequently increase intestinal permeability [62]. Correspondingly, Oliva et al. [63] showed that COVID-19 patients displayed a high level of microbial translocation and increased gut permeability. Together, these evidence suggested that the decrease of anti-inflammatory butyrate-producing bacteria and enrichment of pro-inflammatory bacteria in COVID-19 patients during the acute phase might disturb intestinal barrier function and precipitate microbial translocation, which may further drive immune dysfunction [53].

The alpha diversity indices of gut microbiota in COVID-19 recovered patients were significantly lower than that of non-COVID-19 individuals, which implied that the gut microbiota of COVID-19 recovered patients were still in unhealthy ecostates. Nevertheless, the diversity showed an increase trend in recovered patients compared to COVID-19 patients during the acute phase, suggesting the unhealthy ecostates progressed towards the healthy ecostate after clearance of SARS-CoV-2. Differential abundance analyses revealed that the composition of gut microbiota in recovered patients was broadly closed to that of COVID-19 patients in the acute phase, but different from that of non-COVID-19 individuals to a certain extent. Zhang et al. [55] revealed that gut microbiota in COVID-19 patients manifested lasting impairment of SCFAs and L-isoleucine biosynthesis after disease resolution. Correspondingly, a high proportion of COVID-19 patients exhibited long-term immunologic effects after discharge [64]. Gut dysbiosis that persisted even after clearance of SARS-CoV-2, may be along with persistent gut barrier dysfunction and increased microbial translocation, driving systemic inflammation and promoting long-term immunologic effects in COVID-19 patients. Given the clinical relevance of microbiome disruption in patients with prolonged illness [65], the association between intestinal microbiome disruption and long COVID-19 needs further validation. Probiotics have been proposed for COVID-19 [16, 17, 50, 66]; however, most probiotics currently used are commonly beneficial to multiple diseases. Specific and disease-oriented probiotics are urgently needed, which can effectively promote the health and provide insight into how microbiomes may have restored health [67–69]. Moreover, approaches aimed to restore impaired gut barrier function in COVID-19 patients might be valuable therapeutic strategies.

This study has several limitations. First, we could not include medications and gastrointestinal symptoms during the acute phase and other demographic factors as covariates in the GAMLSS-BEZI model due to insufficient metadata. Consequently, it was not possible to assess these factors on gut microbiota of COVID-19 patients. A more robust cohort of healthy volunteers from whom a baseline sample before COVID-19 diagnosis and then follow-up samples before and after medications in case of COVID-19 diagnosis are collected, would allow deeper investigation of COVID-specific effects on the gut mocrobiota. Our meta-analysis integrated findings of the original studies since most of these studies were conducted without adjusting for any relevant covariates. Second, several studies included in our meta-analysis were small in size, especially studies involved recovered patients, due to the difficulty in recruiting of recovered patients after discharge. However, sensitivity analyses that removing either of the included studies imply that our results are robust. Further, in order to examine whether the differences were driven entirely by Chinese studies, we conducted sensitivity analyses in which removing Chinese studies in the differential abundance analysis of COV vs. non-COV, and the results did not change substantially. Third, since only a few studies collected the information of disease severity, there may be bias in our results of overall non-severe vs. severe analysis. To determine how exactly disease severity might affect gut microbiota in COVID-19, further large sample studies with more focus on different disease severity levels are suggested.

In conclusion, our meta-analysis reported a dysbiotic gut bacterial profile in COVID-19 patients during the acute phase, characterized by depletion of anti-inflammatory butyrate-producing bacteria and enrichment of taxa with pro-inflammatory properties. In addition, gut dysbiosis persisted even after clearance of SARS-CoV-2. Our analysis presents synthesizing knowledge of the current understanding of COVID-19 microbiology based on 16S rRNA gene datasets and provides evidence for future research on the specific COVID-19 associated bacteria. In future work, studies on the specific species of the COVID-19-related bacterial taxa and robust cohort studies with more focus on adjusting for covariates, are suggested to further understand the roles of gut mocrobiota in onset and progression of COVID-19.

## Materials and methods

### Search strategy and selection criteria

A systematic literature review was conducted for relevant studies published prior to May 2022 in PubMed, Web of Science, Embase, Scopus, and Sequence Read Archive (SRA) with keyword and controlled vocabulary terms for COVID-19 and the gut microbiota (see Table S1 for the detailed search strategy). The inclusion criteria for eligible studies of our meta-analysis were as follows: all studies had to (i) be focused on assessing the relationships between the gut microbiota and COVID-19; (ii) the study subjects are ≥18 years old; (iii) use human clinical samples; (iv) include a comparative non-COVID-19 control, unless the study was focused on the gut microbiota of COVID-19 patients with different severity levels; (v) perform 16S rRNA gene sequencing; (vi) have publicly available raw data and corresponding metadata or made available upon request by personal communication with the authors; and (vii) be written in English. Review articles, case reports, or studies without full data available were excluded. Two independent reviewers (X.M.C. and Y.F.L.) assessed each article; differences were resolved by consensus. Since data of our study was publicly available from known publications, patient consent or institutional review board approval was not required.

### Study population sampling

We included only stool samples or rectal swabs. Samples were assigned to different groups before downstream analyses. Designation of the group relied on the information or the metadata provided by the initial study. Samples derived from COVID-19 patients in the acute phase who were detected positive for SARS-CoV-2 RNA were grouped as COV. Samples derived from SARS-CoV-2 non-infected subjects who were healthy individuals or seen by the hospital for unrelated respiratory medical conditions were grouped as non-COV. Samples derived from COVID-19 recovered patients who with clearance of SARS-CoV-2 RNA (negative conversion of viral RNA) within one month were grouped as RP; those with negative conversion of viral RNA more than three months were especially grouped as post-RP.

### Data processing

Raw sequencing data from each included study were processed separately through a standardized pipeline in QIIME2 (Version 2020.6) [70]. After demultiplexing, the DADA2 plugin was used to perform sequence quality control and construct the feature table of amplicon sequence variants (ASVs) [71]. Sequences of mitochondria, or chloroplast, were removed from further analysis. For taxonomic structure analysis, taxonomy was assigned to ASVs using a pre-trained GREENGENES 13_8 99% database and the q2-feature-classifier plugin [72]. Alpha diversity analysis including Shannon’s diversity index, observed species, Pielou’s evenness and Faith’s phylogenetic diversity were calculated using the core-diversity plugin of QIIME2. Prior to the alpha diversity analysis, the feature table of each dataset was subsampled to an even level of coverage. For the available longitudinal sample data, we analyzed only the first sample for the COV group and the last sample for the RP group by date.

### Data synthesis and analysis

Meta-analyses for four measures of alpha diversity were performed utilizing the R package meta and metafor (R version 4.1.0) [73]. Fixed and random effects model estimates were calculated by Hedges’ *g* standardized mean difference statistic; those with 95% confidence intervals (95% CI) above or below 0 were regarded as statistically significant. The *I*^2^ (percentage of variation reflecting true heterogeneity), *τ*^2^ (random-effects between study variance), and *p*-value from Cochran’s *Q* test was used to assess statistically significant heterogeneity. Asymmetry of the funnel plots were applied to detect the publication biases when the number of studies is greater than five. Begg’s correlation test and Egger’s regression test were also used to detect the publication biases when the number of studies is greater than ten, with a *P* value < 0.1 indicating a potential bias [74, 75]. Standardized alpha diversity indexes were calculated through mean centering to zero within each study and scaling to unit variance. Boxplots were generated using Prism 8.3.0 (GraphPad Software).

Counts of bacterial taxa were converted to relative abundances. Relative abundance data were then filtered to retain only the taxa that had an average relative abundance of at least 0.005% and were present in at least 5% of samples in that study. Generalized Additive Models for Location, Scale and Shape (GAMLSS) with a zero-inflated beta distribution (BEZI) implemented in the R package gamlss were used to examine relative abundances of bacterial taxa in each study [76, 77]. To evaluate the overall effects while addressing heterogeneity across studies, we applied random-effects meta-analysis models with inverse variance weighting and DerSimonian–Laird estimator for between-study variance across all included studies to combine the adjusted estimates and their standard errors. Meta-analyses were done for only bacterial taxa whose adjusted estimates and standard errors were available in at least 50% of the number of included studies. To account for the robustness of the results, sensitivity analyses were performed by removing one study at a time. All statistical tests were two-sided. A *P* value of less than 0.05 was considered significant and a false discovery rate (FDR)-adjusted *P* value of less than 0.1 was considered significant after adjusting for multiple testing.

## Supplementary Information

**Additional file 1.** .

## Data Availability

Raw data for this meta-analysis are available online (see Table S8). The R script used for this meta-analysis is included in the Supplementary Text and has been uploaded on https://github.com/XiaominCheng/16S_gut_meta. The authors confirmed that all supporting data have been provided within the article or through supplementary materials.
