# Supplementary material for: Meta-analysis of 16S rRNA microbial data identified alterations of the gut microbiota in COVID-19 patients during the acute and recovery phases
Source: BMC Microbiol. 2022 Nov 14;22:274. doi: 10.1186/s12866-022-02686-9 (PMC9662111; doi:10.1186/s12866-022-02686-9)
Supplement: Supplementary file 1 — Additional file 1. . [file 12866_2022_2686_MOESM1_ESM.pdf]

Supplementary Tables and Figures

Meta-Analysis of 16S rRNA Microbial Data Identified Alterations of the Gut Microbiota in COVID-19 Patients during the Acute and Recovery Phases

Xiaomin Cheng, Yali Zhang, Yifan Li, Qin Wu, Jiani Wu, Soo-Kyung Park, Cheng Guo, and Jiahai Lu

List of Supplementary Tables

Table S1. The search strategy.

Table S2. Characteristics of studies not included in Meta-Analysis.

Table S3. Meta-analysis of all included studies for gut bacterial taxa with differential relative abundances between COV vs. non-COV.

Table S4. Differential abundance analysis between COV vs. non-COV: sensitivity analysis removing the four Chinese studies.

Table S5. Meta-analysis of all included studies for gut bacterial taxa with differential relative abundances between severe vs. non-severe COVID-19 patients.

Table S6. Meta-analysis of all included studies for gut bacterial taxa with differential relative abundances between RP/post-RP vs. non-COV.

Table S7. Meta-analysis of all included studies for gut bacterial taxa with differential relative abundances between RP/post-RP vs. COV.

Table S8. The raw data details

List of Supplementary Figures

Fig. S1 Flowchart for study search and selection.

Fig. S2 Forest plot of the differences in alpha diversity between COV and non-COV by (a) Pielou's evenness, and (b) Faith's phylogenetic diversity.

Fig. S3 Sensitivity analysis for the differences in alpha diversity between COV and non-COV after the removal of either of the included studies.

Fig. S4 Funnel plot for publication for the differences in alpha diversity between COV and non-COV.

Fig. S5 Forest plot of the differences in alpha diversity between severe and non-severe COVID-19 patients by (a) Shannon’s diversity index, (b) observed species, (c) Pielou’s evenness, and (d) Faith’s phylogenetic diversity.

Fig. S6 Forest plot of the differences in alpha diversity between RP/post-RP and non-COV by (a) Pielou’s evenness, and (b) Faith’s phylogenetic diversity.

Fig. S7 Sensitivity analysis for the differences in alpha diversity between RP/post-RP and non-COV after the removal of either of the included studies.

Fig. S8 Funnel plot for publication for the differences in alpha diversity between RP/post-RP and non-COV.

Fig. S9 Test for subgroup differences demonstrated no significance when studies stratified by RP and post-RP in alpha diversity.

Fig. S10 Boxplots showing standardized alpha diversity by SARS-CoV-2 infection status.

Fig. S11 Differential abundance analysis between COV vs. non-COV (a) at genus level, and (b) at species level: sensitivity analysis removing the four Chinese studies.

Table S1. The search strategy

Information sources	Search terms
**PubMed**	("COVID-19"[Title/Abstract] OR "coronavirus disease 19"[Title/Abstract] OR "SARS-CoV-2"[Title/Abstract] OR "severe acute respiratory syndrome coronavirus 2"[Title/Abstract]) AND ("microbio*"[Title/Abstract] OR "microflora*"[Title/Abstract] OR "bacteria*"[Title/Abstract] OR "microbiome*"[Title/Abstract] OR "flora*"[Title/Abstract] OR "bacterial*"[Title/Abstract] OR "microorganism"[Title/Abstract] OR "dysbiosis"[Title/Abstract]) AND ("gut"[All Fields] OR "intestine*"[All Fields] OR "bowel*"[All Fields] OR "gastrointestinal*"[All Fields] OR "feces*"[All Fields] OR "stool*"[All Fields] OR "rectal*"[All Fields]) AND "16S"[All Fields]
**Web of Science**	(TS=(COVID-19 OR “Coronavirus Disease 19” OR SARS-CoV-2 OR “Severe Acute Respiratory Syndrome Coronavirus 2” OR 2019-nCoV OR “2019 Novel Coronavirus”) AND TS=(microbiota* OR microflora* OR bacteria* OR microbiome* OR flora* OR bacterial* OR bacteria* OR microorganism OR dysbiosis) AND TS=(gut* OR intestine* OR bowel* OR gastrointestinal* OR feces* OR stool* OR rectal*) AND TS=(16S))
**Embase**	('covid-19' OR 'coronavirus disease 19' OR 'sars-cov-2' OR 'severe acute respiratory syndrome coronavirus 2' OR '2019 ncov' OR '2019 novel coronavirus') AND (microbiota* OR microflora* OR microbiome* OR flora* OR bacterial* OR bacteria* OR microorganism OR dysbiosis) AND (gut* OR intestine* OR bowel* OR gastrointestinal* OR feces* OR stool* OR rectal*) AND 16s
**Scopus**	(TITLE-ABS-KEY("COVID-19" OR "Coronavirus Disease 19" OR "SARS-CoV-2" OR "Severe Acute Respiratory Syndrome Coronavirus 2" OR "2019 nCoV" OR "2019 Novel Coronavirus") AND TITLE-ABS-KEY("microbiota*" OR "microflora*" OR "bacteria*" OR "microbiome*" OR "flora*" OR "bacterial*" OR "bacteria*" OR "microorganism" OR "dysbiosis") AND ALL(gut* OR intestine* OR bowel* OR gastrointestinal* OR feces* OR stool* OR rectal*) AND ALL("16S")) AND (EXCLUDE (DOCTYPE,"re")) AND (LIMIT-TO (LANGUAGE,"English"))
**SRA**	(“COVID-19” OR “SARS-CoV-2”) [AND] (microbiota* OR microflora* OR bacteria* OR microbiome* OR flora* OR bacterial* OR bacteria* OR microorganism OR dysbiosis) [AND] (gut* OR intestine* OR bowel* OR gastrointestinal* OR feces* OR stool*)

Table S2. Characteristics of studies not included in Meta-Analysis

Author, Year [Ref]	Country	Design	Study Population	Sample Type	16S region	Sequencing Platform	Differential analysis	Reasons for exclusion
Jagielski 2022 [34]	Poland	Case-control	8 COV, 14 non-COV	Fecal samples	NM	NM	NM	Raw data cannot be obtained
Junior 2022 [35]	Brazil	Case-control	149 post-COV, 70 non-COV	Fecal samples	V3-V4	MiSeq	corncob	Unpublished and disease status is ambiguity
Mizutani 2022 [36]	Japan	Case-control	22 COV (39 samples), 40 non-COV, 6 RP	Fecal samples	V3–V4	MiSeq	LEfSe	Unable to distinguish the raw data of COV and RP
Schult 2022 [37]	Germany	Case-control	107 COV (210 samples), 37 non-COV (41 samples)	Fecal samples	V3–V4	MiSeq	adonis	Disease status was not reported in the metadata
Wu 2021 [17]	China	Case-control	13 COV, 30 non-COV (15 HCs,15 NC-PCs)	Fecal samples	V3–V4	MiSeq	Wilcoxon	Raw data cannot be obtained
Venzon 2021 [38]	USA	Cross-sectional	101 COV	Fecal samples	V4	MiSeq	NM	Lacked non-COV
Xu 2021 [39]	China	Cross-sectional	19 COV	Fecal samples	V4	MiSeq	NM	Lacked non-COV

NM, not mentioned; HCs, healthy controls; NC-PCs, non-COVID-19 pneumonia controls.

Table S3. Meta-analysis of all included studies for gut bacterial taxa with differential relative abundances between COV vs. non-COV.

**Bacterial taxa** [a]	**Pooled estimate(log(OR))**	**95% Pooled lower limit**	**95% Pooled upper limit**	**Pooled p-value**	**FDR adjusted pooled p-value**
f__Ruminococcaceae.g__Ruminococcus	-0.33	-0.48	-0.19	0	2.00E-04
f__Lachnospiraceae.g__Lachnospira	-0.63	-0.82	-0.44	0	0
f__Streptococcaceae.g__Streptococcus	0.43	0.24	0.63	0	3.00E-04
f__Ruminococcaceae.g__Faecalibacterium	-0.6	-0.93	-0.26	5.00E-04	0.0063
f__Lachnospiraceae.g__Coprococcus	-0.52	-0.81	-0.23	5.00E-04	0.0063
f__Lachnospiraceae.g__Roseburia	-0.58	-0.92	-0.23	0.001	0.0093
f__Prevotellaceae.g__Prevotella	-0.4	-0.64	-0.16	0.001	0.0093
f__Veillonellaceae.g__Dialister	-0.48	-0.79	-0.17	0.0025	0.0203
f__Veillonellaceae.g__Megasphaera	-0.52	-0.87	-0.18	0.0029	0.0204
f__Pasteurellaceae.g__Haemophilus	-0.34	-0.57	-0.1	0.0049	0.0311
f__Veillonellaceae.g__Megamonas	-0.4	-0.68	-0.12	0.0056	0.0326
f__Erysipelotrichaceae.g__Catenibacterium	-0.46	-0.85	-0.07	0.0202	0.0931
f__Paraprevotellaceae.g__Paraprevotella	-0.28	-0.51	-0.04	0.0203	0.0931
f__Enterococcaceae.g__Enterococcus	0.33	0.05	0.6	0.0204	0.0931
f__Corynebacteriaceae.g__Corynebacterium	0.4	0.06	0.75	0.0222	0.0946
f__Turicibacteraceae.g__Turicibacter	-0.33	-0.62	-0.04	0.0269	0.1075
f__Erysipelotrichaceae.g__Bulleidia	-0.59	-1.12	-0.06	0.0295	0.1109
f__Veillonellaceae.g__Phascolarctobacterium	-0.21	-0.41	-0.02	0.0315	0.112
f__Coriobacteriaceae.g__Eggerthella	0.21	0	0.41	0.0454	0.1531
g__Faecalibacterium.s__prausnitzii	-0.52	-0.83	-0.22	8.00E-04	0.0507
g__Prevotella.s__copri	-0.45	-0.74	-0.17	0.0017	0.0523
g__Clostridium.s__lavalense	0.33	0.08	0.58	0.0098	0.1462
g__Bifidobacterium.s__adolescentis	-0.22	-0.38	-0.05	0.0101	0.1462

Table S3. Continued

**Bacterial taxa** [a]	**Pooled estimate(log(OR))**	**95% Pooled lower limit**	**95% Pooled upper limit**	**Pooled p-value**	**FDR adjusted pooled p-value**
g__Bacteroides.s__caccae	0.18	0.04	0.32	0.0143	0.1462
g__Eubacterium.s__dolichum	0.26	0.05	0.47	0.0166	0.1462
g__Haemophilus.s__parainfluenzae	-0.28	-0.51	-0.05	0.0191	0.1462
g__Ruminococcus.s__bromii	-0.30	-0.55	-0.05	0.0192	0.1462
g__Streptococcus.s__anginosus	0.34	0.03	0.65	0.0297	0.2012
g__Roseburia.s__faecis	-0.34	-0.66	-0.01	0.0411	0.2316
g__Clostridium.s__spiroforme	0.55	0.02	1.08	0.0418	0.2316
g__Veillonella.s__dispar	0.19	0	0.37	0.0479	0.2437

[a] Only bacterial taxa with a pooled p-value below 0.05 were displayed.

Table S4. Differential abundance analysis between COV vs. non-COV: sensitivity analysis removing the four Chinese studies.

Bacterial taxa [a]	Pooled estimate(log(OR))	95% Pooled lower limit	95% Pooled upper limit	Pooled p-value	FDR adjusted pooled p-value
f__Lachnospiraceae.g__Lachnospira	-0.62	-0.87	-0.38	0	0
f__Prevotellaceae.g__Prevotella	-0.51	-0.8	-0.21	7.00E-04	0.0236
f__Pasteurellaceae.g__Haemophilus	-0.48	-0.79	-0.17	0.0026	0.049
f__Pseudomonadaceae.g__Pseudomonas	-1.16	-1.93	-0.38	0.0033	0.049
f__Ruminococcaceae.g__Oscillospira	-0.3	-0.51	-0.1	0.0041	0.049
f__Paraprevotellaceae.g__Paraprevotella	-0.4	-0.68	-0.13	0.0041	0.049
f__Ruminococcaceae.g__Ruminococcus	-0.25	-0.43	-0.08	0.0053	0.0508
f__Alcaligenaceae.g__Sutterella	-0.36	-0.61	-0.1	0.0057	0.0508
f__Ruminococcaceae.g__Faecalibacterium	-0.61	-1.12	-0.11	0.0169	0.1335
f__Streptococcaceae.g__Streptococcus	0.3	0.03	0.56	0.0292	0.1902
f__Erysipelotrichaceae.g__Bulleidia	-0.59	-1.12	-0.06	0.0295	0.1902
f__Veillonellaceae.g__Megasphaera	-0.45	-0.87	-0.02	0.0382	0.1993
f__Veillonellaceae.g__Megamonas	-0.49	-0.95	-0.02	0.0392	0.1993
f__Enterococcaceae.g__Enterococcus	0.33	0.01	0.65	0.0413	0.1993
f__Veillonellaceae.g__Dialister	-0.43	-0.85	-0.02	0.0421	0.1993
f__Corynebacteriaceae.g__Corynebacterium	0.4	0.01	0.79	0.0454	0.2017
g__Prevotella.s__copri	-0.56	-0.91	-0.21	0.0019	0.1128
g__Ruminococcus.s__albus	-1.05	-1.92	-0.17	0.0187	0.3392
g__Faecalibacterium.s__prausnitzii	-0.52	-0.96	-0.08	0.0216	0.3392
g__Clostridium.s__celatum	-0.24	-0.45	-0.03	0.0275	0.3392
g__Bifidobacterium.s__adolescentis	-0.19	-0.36	-0.02	0.0283	0.3392
g__Haemophilus.s__parainfluenzae	-0.34	-0.66	-0.01	0.0441	0.4414

[a] Only bacterial taxa with a pooled p-value below 0.05 were displayed.

Table S5. Meta-analysis of all included studies for gut bacterial taxa with differential relative abundances between severe vs. non-severe COVID-19 patients.

Bacterial taxa [a]	Pooled estimate(log(OR))	95% Pooled lower limit	95% Pooled upper limit	Pooled p-value	FDR adjusted pooled p-value
f__Lachnospiraceae.g__Roseburia	-0.31	-0.57	-0.05	0.0184	0.9621
f__Ruminococcaceae.g__Faecalibacterium	-0.49	-0.98	-0.01	0.0456	0.9621
g__Coprococcus.s__eutactus	-0.22	-0.22	-0.21	0	0

[a] Only bacterial taxa with a pooled p-value below 0.05 were displayed.

Table S6. Meta-analysis of all included studies for gut bacterial taxa with differential relative abundances between RP/post-RP vs. non-COV.

Bacterial taxa [a]	Pooled estimate(log(OR))	95% Pooled lower limit	95% Pooled upper limit	Pooled p-value	FDR adjusted pooled p-value
f__Ruminococcaceae.g__Ruminococcus	-0.49	-0.72	-0.26	0	6.00E-04
f__Ruminococcaceae.g__Faecalibacterium	-0.75	-1.05	-0.44	0	0
f__Lachnospiraceae.g__Roseburia	-0.63	-0.87	-0.38	0	0
f__Lachnospiraceae.g__Coprococcus	-0.72	-1.09	-0.36	1.00E-04	0.0014
f__Fusobacteriaceae.g__Fusobacterium	0.7	0.29	1.1	7.00E-04	0.0084
f__Streptococcaceae.g__Streptococcus	0.34	0.08	0.59	0.0092	0.0893
f__Coriobacteriaceae.g__Collinsella	-0.36	-0.67	-0.06	0.0206	0.1708
f__Erysipelotrichaceae.g__Clostridium	0.39	0.01	0.76	0.042	0.2761
f__Pasteurellaceae.g__Haemophilus	0.35	0	0.69	0.0471	0.2761
g__Faecalibacterium.s__prausnitzii	-0.72	-1.02	-0.42	0	1.00E-04
g__Parabacteroides.s__distasonis	0.49	0.18	0.8	0.0019	0.0563
g__Coprococcus.s__eutactus	-1.23	-2.09	-0.38	0.0048	0.095
g__Clostridium.s__hathewayi	0.84	0.21	1.48	0.0093	0.1163
g__Clostridium.s__citroniae	0.54	0.13	0.95	0.0103	0.1163
g__Clostridium.s__ramosum	0.38	0.08	0.67	0.0118	0.1163
g__Prevotella.s__stercorea	-0.87	-1.6	-0.15	0.0184	0.155
g__Ruminococcus.s__gnavus	0.27	0.03	0.52	0.0268	0.1974
g__Roseburia.s__faecis	-0.28	-0.55	-0.01	0.039	0.2556

[a] Only bacterial taxa with a pooled p-value below 0.05 were displayed.

Table S7. Meta-analysis of all included studies for gut bacterial taxa with differential relative abundances between RP/post-RP vs. COV.

**Bacterial taxa** [a]	**Pooled estimate(log(OR))**	**95% Pooled lower limit**	**95% Pooled upper limit**	**Pooled p-value**	**FDR adjusted pooled p-value**
f__Actinomycetaceae.g__Actinomyces	0.39	0.12	0.67	0.0055	0.1972
f__Clostridiaceae.g__SMB53	-0.76	-1.31	-0.22	0.0058	0.1972
f__Enterobacteriaceae.g__Citrobacter	0.84	0.04	1.64	0.0406	0.7727
g__Bifidobacterium.s__breve	1.55	1.38	1.72	0	0
g__Clostridium.s__clostridioforme	-0.77	-1.19	-0.36	3.00E-04	0.0081
g__Ruminococcus.s__lactaris	-0.72	-1.38	-0.05	0.0354	0.6093
g__Bacteroides.s__plebeius	-0.54	-1.06	-0.01	0.0448	0.6093

[a] Only bacterial taxa with a pooled p-value below 0.05 were displayed.

Table S8. The raw data details

Author, Year	Accession number
Al 2021	PRJNA769052
Cervino 2022	PRJNA787810
Chen 2022	PRJNA703303
Gaibani 2021	PRJNA700830, mgp17761, PRJNA247489
Gu 2020	PRJNA636824
Kim 2021	— [a]
Khan 2021	PRJNA705797
Mazzarelli 2021	PRJNA681516
Moreira-Rosario 2021	PRJNA734646
Newsome 2021	PRJNA678695
Rafiqul 2022	PRJNA767939
Reinold 2021	PRJNA747262
Ren 2021	PRJNA660302
Tian 2021	PRJNA683685
Wu 2021	PRJNA684070
Zhou 2021	PRJNA736160

[a] The raw sequencing data are available on reasonable request from the corresponding author of Kim 2021

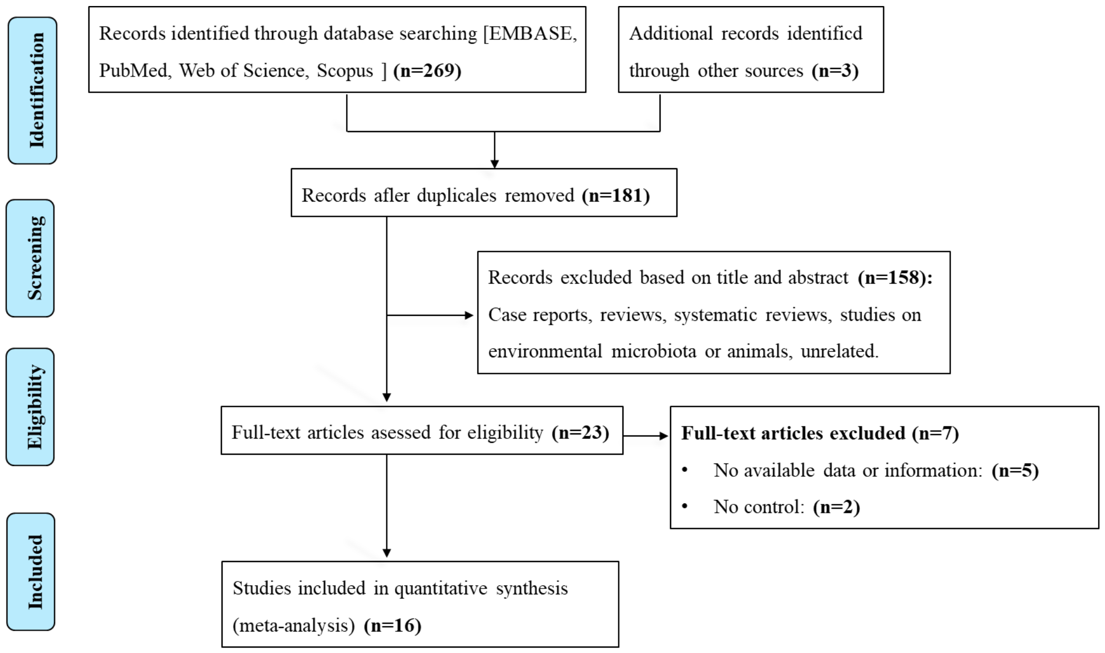

Fig. S1 Flowchart for study search and selection.

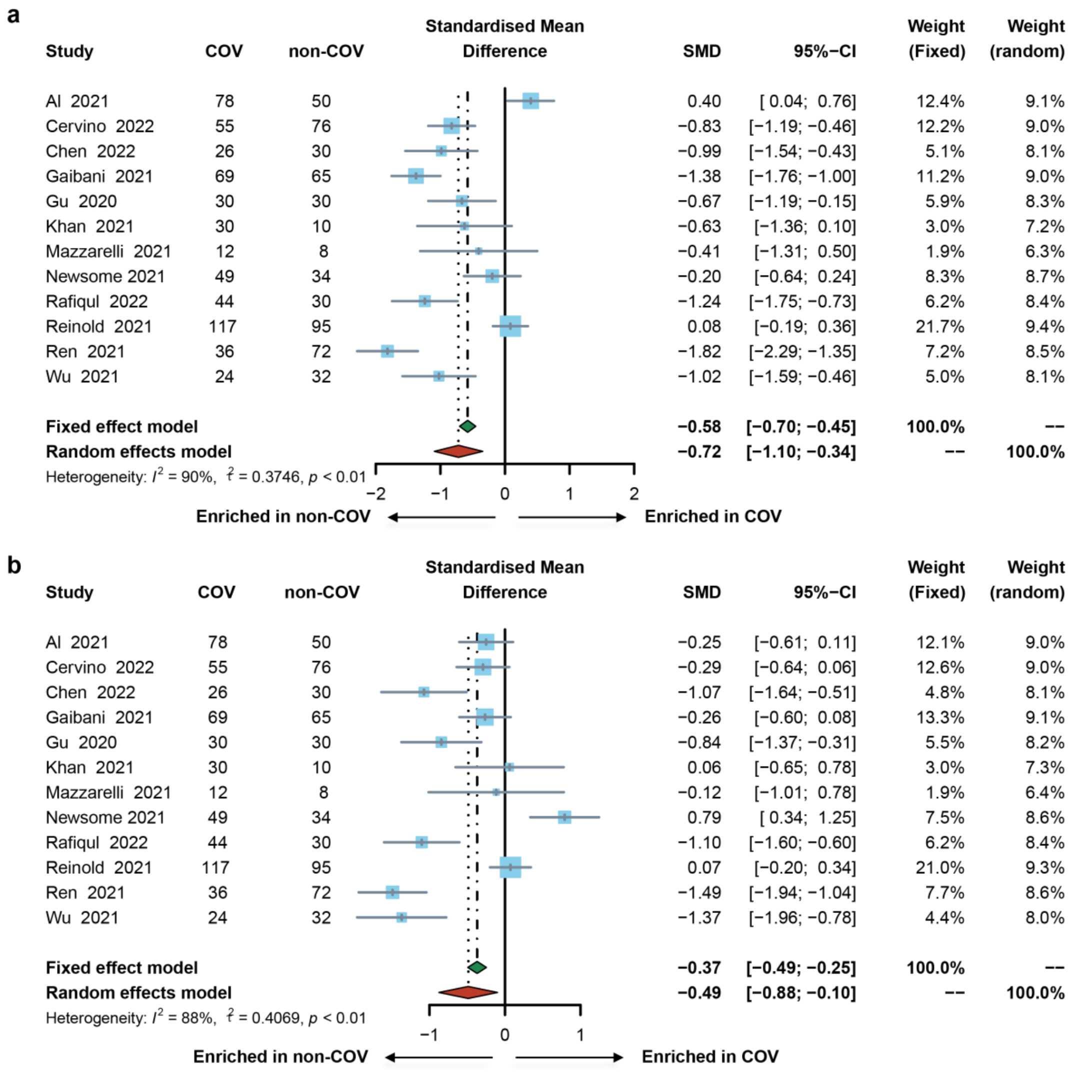

Fig. S2 Forest plot of the differences in alpha diversity between COV and non-COV by (a) Pielou's evenness, and (b) Faith's phylogenetic diversity.

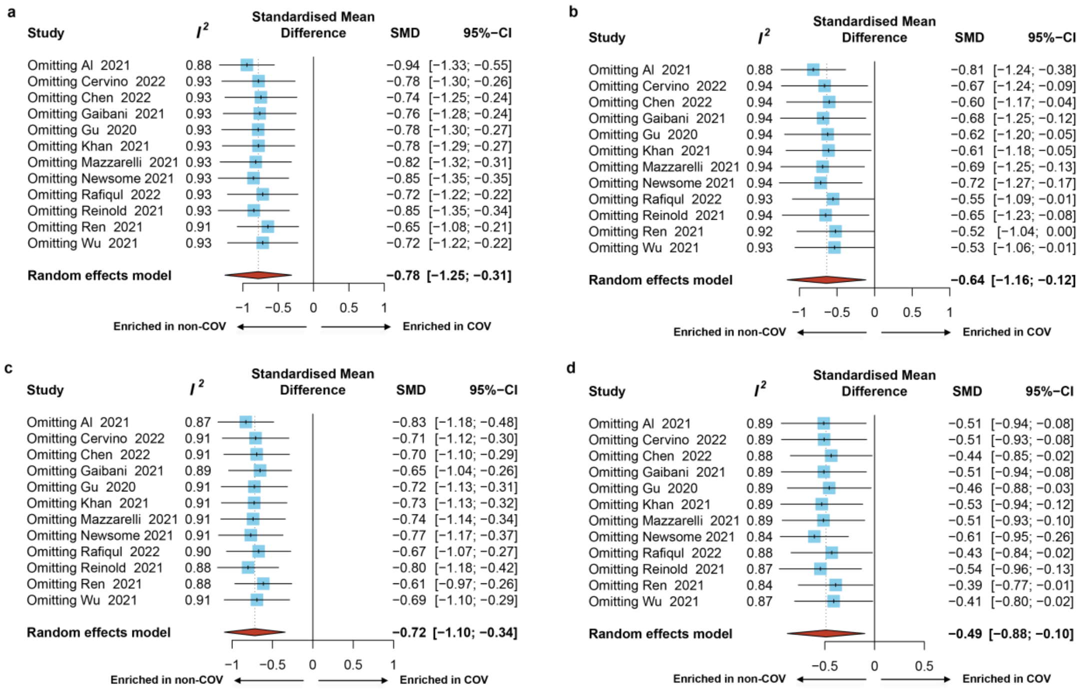

Fig. S3 Sensitivity analysis for the differences in alpha diversity between COV and non-COV after the removal of either of the included studies. (a) Shannon's diversity index, (b) observed species, (c) Pielou's evenness, and (d) Faith's phylogenetic diversity. Random-effects models (red diamonds) with 95% CI above or below 0 were considered statistically significant.

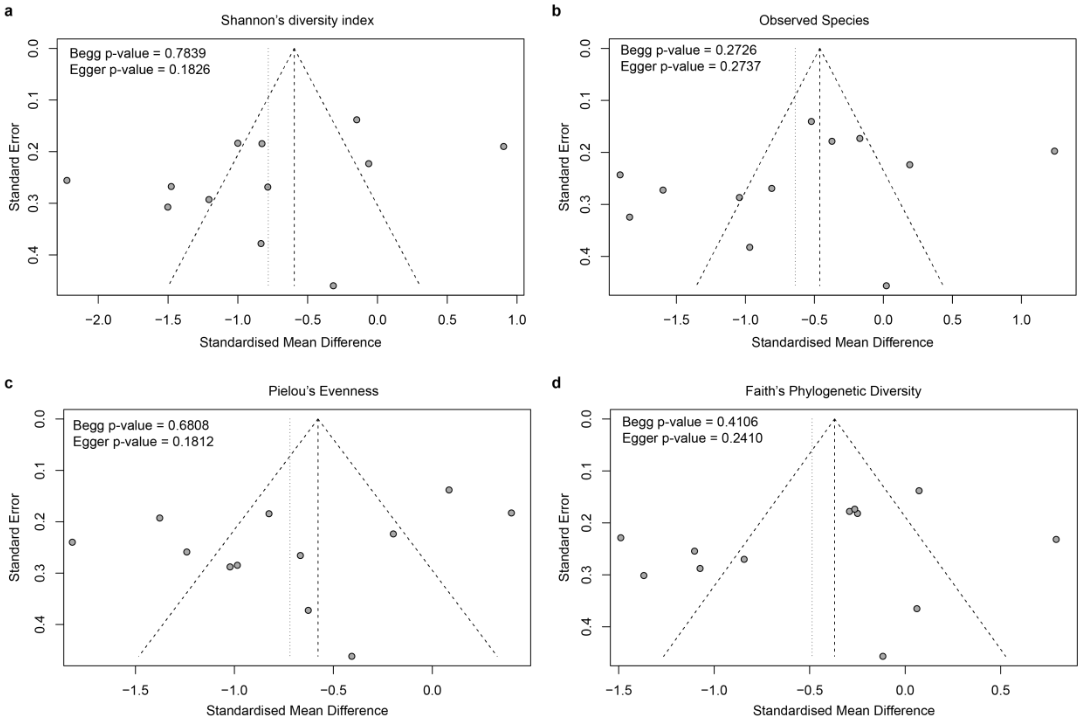

Fig. S4 Funnel plot for publication for the differences in alpha diversity between COV and non-COV. (a) Shannon's diversity index, (b) observed species, (c) Pielou's evenness, and (d) Faith's phylogenetic diversity. Begg's correlation test and Egger's regression test were also used to detect the publication biases, with a *P* value < 0.1 indicating a potential bias.

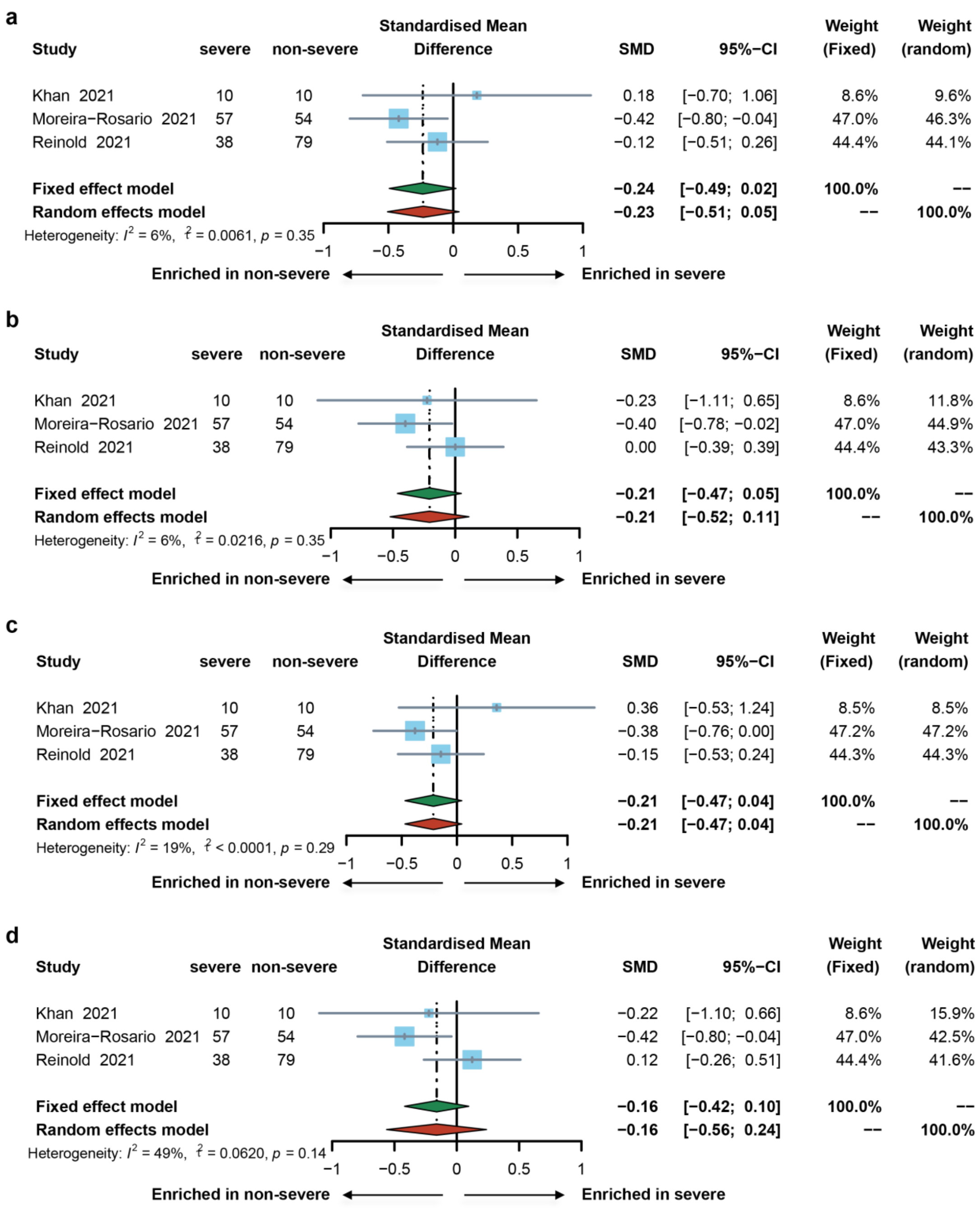

Fig. S5 Forest plot of the differences in alpha diversity between severe and non-severe COVID-19 patients by (a) Shannon's diversity index, (b) observed species, (c) Pielou's evenness, and (d) Faith's phylogenetic diversity.

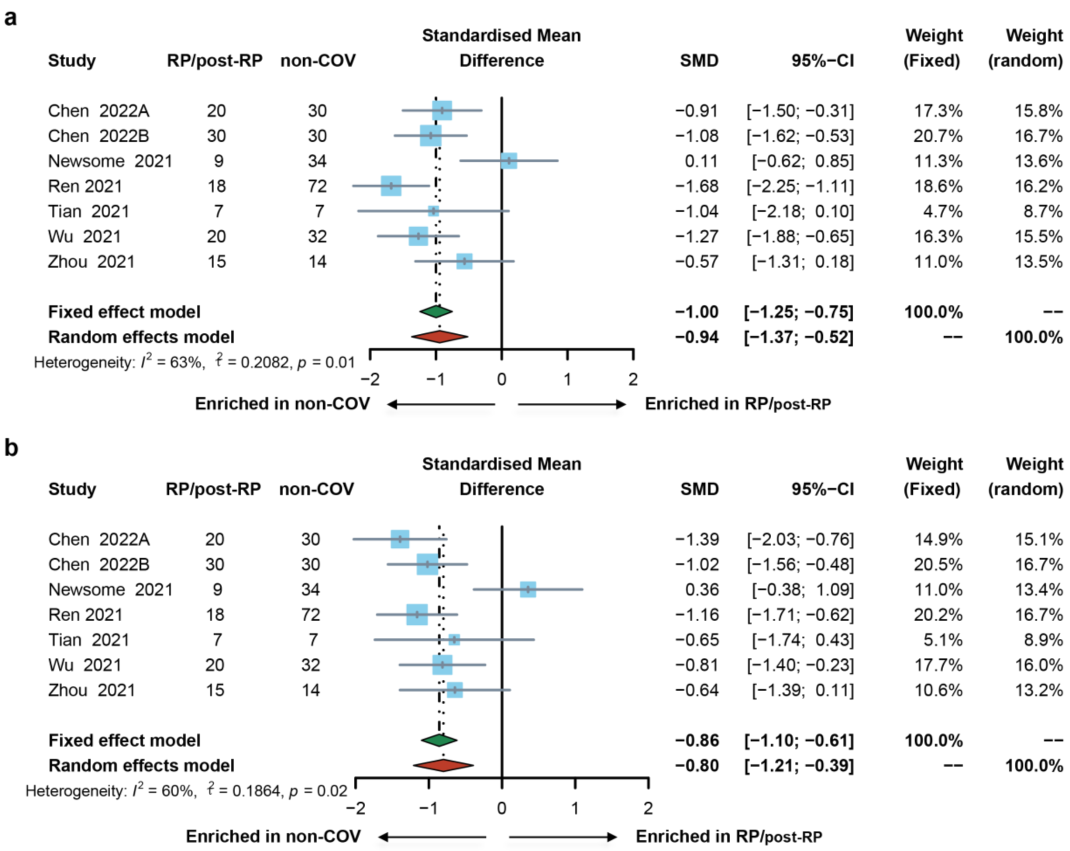

Fig. S6 Forest plot of the differences in alpha diversity between RP/post-RP and non-COV by (a) Pielou's evenness, and (b) Faith's phylogenetic diversity.

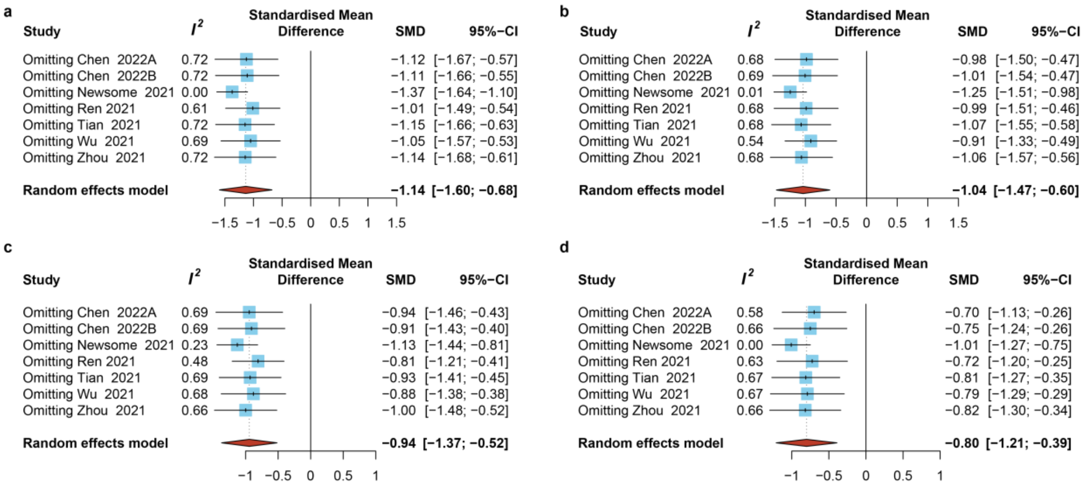

Fig. S7 Sensitivity analysis for the differences in alpha diversity between RP/post-RP and non-COV after the removal of either of the included studies. (a) Shannon's diversity index, (b) observed species, (c) Pielou's evenness, and (d) Faith's phylogenetic diversity. Random-effects models (red diamonds) with 95% CI above or below 0 were considered statistically significant.

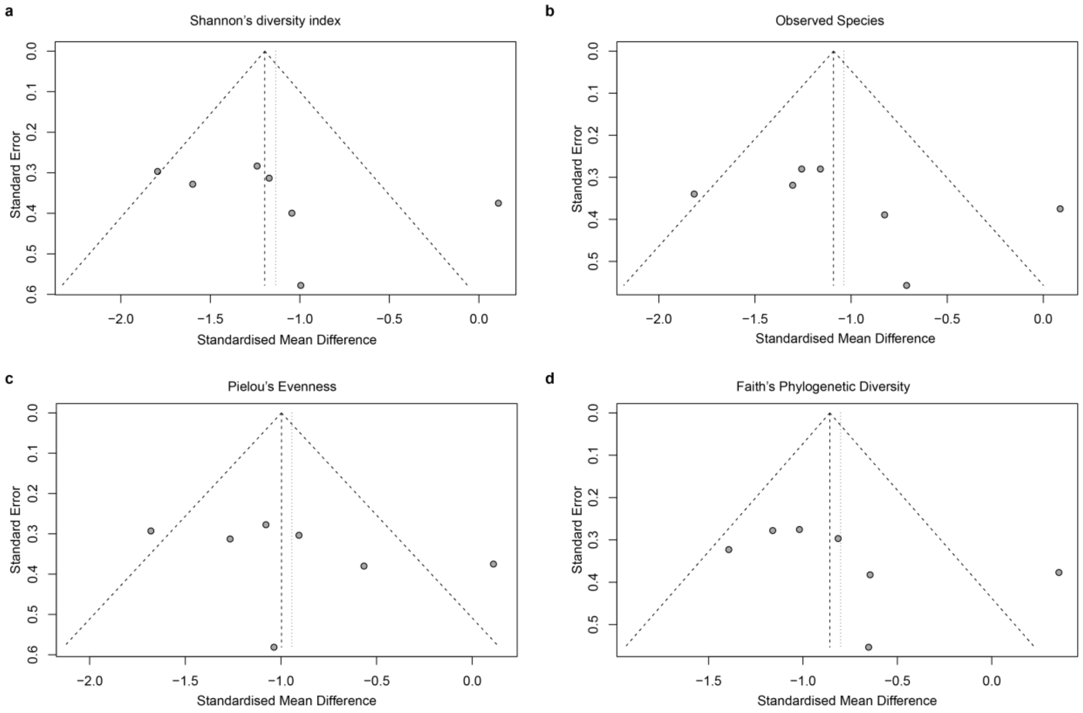

Fig. S8 Funnel plot for publication for the differences in alpha diversity between RP/post-RP and non-COV. (a) Shannon's diversity index, (b) observed species, (c) Pielou's evenness, and (d) Faith's phylogenetic diversity.

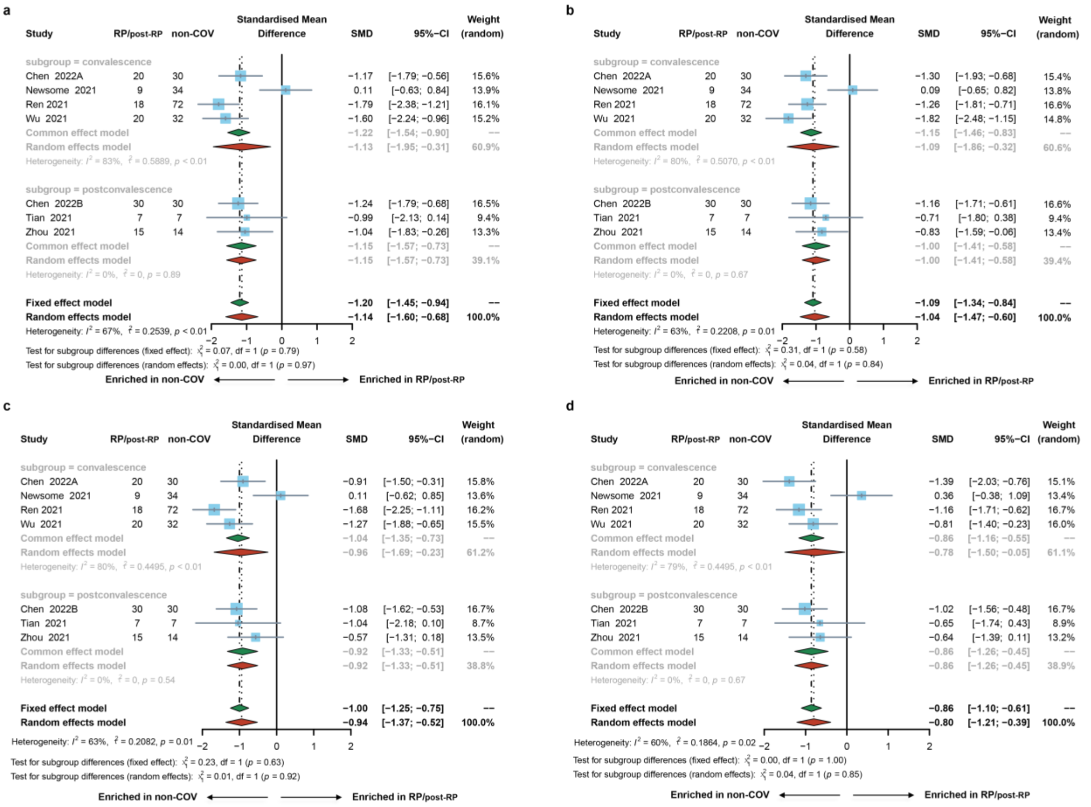

Fig. S9 Tests for subgroup differences demonstrated no significance when studies stratified by RP and post-RP in alpha diversity. (a) Shannon's diversity index, (b) observed species, (c) Pielou's evenness, and (d) Faith's phylogenetic diversity. The random effects model results were emphasized.

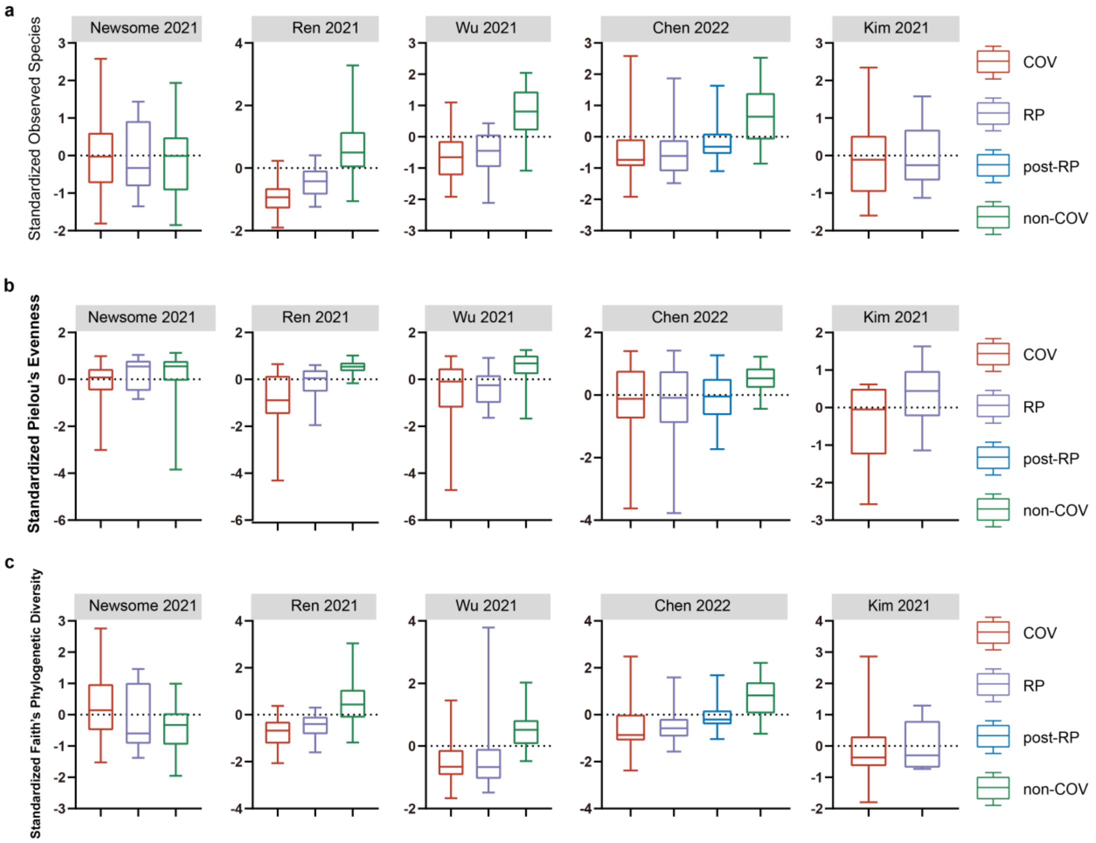

Fig. S10 Boxplots showing standardized alpha diversity by SARS-CoV-2 infection status. (a) Standardized observed species, (b) Standardized Pielou's evenness, and (c) Standardized Faith's phylogenetic diversity. Red = COV, purple = RP, blue = post-RP, and green = non-COV.

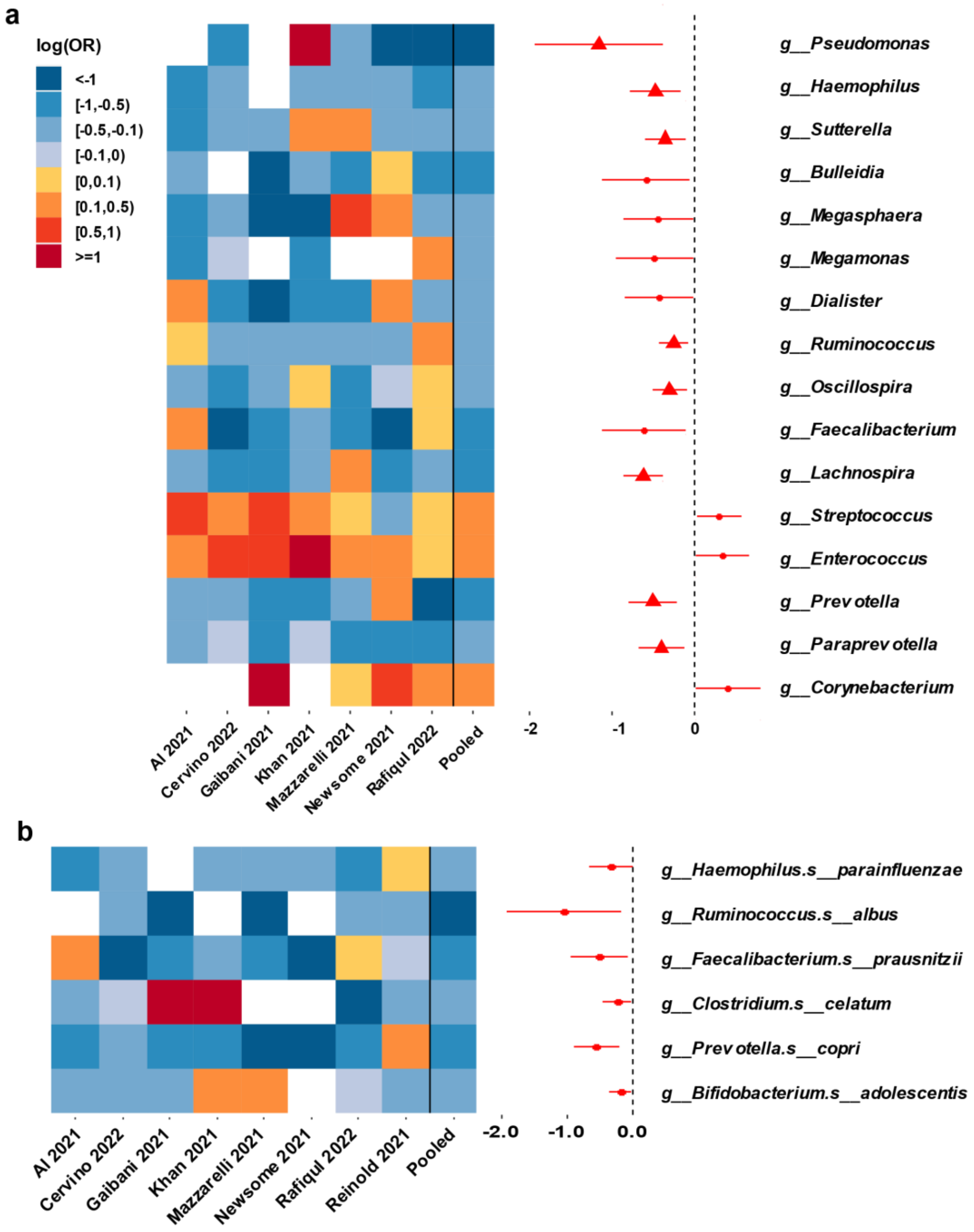

Fig. S11 Differential abundance analysis between COV vs. non-COV (a) at genus level, and (b) at species level: sensitivity analysis removing the four Chinese studies. Heatmap showed log(OR) of relative abundances of bacterial taxa between COV and non-COV across each study. The bacterial taxa unavailable in a particular study were in white in heatmap. Forest plot indicated pooled log(OR) estimate and 95% CI of relative abundances of bacterial taxa between COV and non-COV across all studies included. Log(OR) estimates were from GAMLSS-BEZI and Random Effects Meta-analysis. Only bacterial taxa with pooled *P* of pooled log(OR) estimates below 0.05 were displayed. Pooled log(OR) estimates with FDR-adjusted pooled *P* <0.1 were showed as red triangles. Log(OR) > 0 denoted an increase and log(OR) < 0 denoted a decrease of taxa relative abundance in COV compared with non-COV.
